# In search of traces of the mandrake myth: the historical, and ethnobotanical roots of its vernacular names

**DOI:** 10.1186/s13002-021-00494-5

**Published:** 2021-12-04

**Authors:** Amots Dafni, Cesar Blanché, Salekh Aqil Khatib, Theodora Petanidou, Bedrettin Aytaç, Ettore Pacini, Ekaterina Kozuharova, Aharon Geva-Kleinberger, Soli Shahvar, Zora Dajic, Helmut W. Klug, Guillermo Benítez

**Affiliations:** 1 Department of Evolutionary and Environmental Biology and Institute of Evolution, University of Haifa, Haifa, Israel; 2 GREB-BioC, Botany Laboratory, Faculty of Pharmacy and Food Sciences, University of Barcelona, Av. Joan XXIII S/N, 08028 Barcelona, Catalonia, Spain; 3 20128 Mghar, Israel; 4 Laboratory of Biogeography and Ecology, Department of Geography, University of the Aegean, 81100 Mytilene, Greece; 5 Department of Arabic Language and Literature, Faculty of Languages, History and Geography, Ankara University, Ankara, Turkey; 6 Department of Life Sciences, Università degli Studi di Siena, Siena, Italy; 7 Faculty of Pharmacy, Department of Pharmacognosy, Medical University of Sofia, Dunav 2 sr., 1000 Sofia, Bulgaria; 8 Department of Arabic Language and Literature, University of Haifa, Haifa, Israel; 9 Department of Middle Eastern and Islamic Studies, The Ezri Center for Iran and Persian Gulf Studies, The University of Haifa, Haifa, Israel; 10 Faculty of Agriculture, Department of Applied Botany, University of Belgrade, Nemanjina 6, 11080 Belgrade, Republic of Serbia; 11 Centre for Information Modelling, University of Graz, Graz, Austria; 12 Department of Botany, University of Granada, Campus Universitario de Cartuja, 18071 Granada, Spain

**Keywords:** *Mandragora* spp., Plant names, Etymology, Phytonymy

## Abstract

**Background:**

Mandrake (*Mandragora* spp.) is one of the most famous medicinal plant in western cultures since Biblical times and throughout written history. In many cultures, mandrake is related to magic and witchcraft, which is said to have a psychosomatic effect (especially when mandrake contains narcotic compounds) in addition to the pharmacological influence, as occurs with other narcotic magical plants. Due to its unique properties and related myths, it is not surprising that this plant has many names in many languages.

**Methods:**

This paper presents an attempt to reconstruct the historical, ethnobotanical, and folkloristic roots of 292 vernacular names of *Mandragora* spp. in forty-one languages. We used the plant’s morphological data, philology, myths and legends, medicinal properties and uses, as well as historical evidence and folkloric data, to explain meaning, origin, migration, and history of the plant’s names.

**Results:**

The names were classified into the following main categories: Derivatives of *mandragora* (19 languages), *alraun* (7) and of *yabroukh* (5). The salient groups of the plant’s vernacular names are related to: Anthropomorphism (33 names in 13 languages); Similarity to other plants (28/9); Supernatural agents (28/9); Narcotic effects (21/8); Leaves, fruits, and seeds (21/8); Aphrodisiac properties (17/10); Use of a dog (15/9); Gallows (14/5); Black magic, sorcery, witchcraft (13/8), and Medicinal use (11/7).

**Conclusions:**

This frequency distribution of the mandrake’s vernacular names reflects its widespread reputation as related to the doctrine of signatures, beliefs in its supernatural, natural, and mythic powers, and to a lesser extent, its uses in magic and medicine. A spatiotemporal analysis of the mandrake’s names supports the old idea that the pulling ceremonies for this plant originated in the Near East and that various other myths related to this plant may have originated in different places and periods.

## Introduction

Gledhill [1:1–2] noted that “Common plant names present language at its richest and most imaginative.… Local variations in common names are numerous and this is perhaps a reflection of the importance of the plant in general conversation in the kitchen and in herbalism throughout the country in bygone days.”

Some names refer unequivocally to a specific plant species (monosemic name), while other names can be used for different plants (polysemic names). The proliferation of names for individual botanical species is related to a variety of factors: the geographical range of the plant and languages spoken in its area, the ethnobotanical value as a ritual and/or medicinal plant, its strange appearance or resemblance to familiar objects, etc.). For example, *Pistacia terebinthus* L. has a variety of names throughout Greece, which probably reflects the importance of the plant for local societies [2, 3]. Functionality, however, is no guarantee of name diversity. Consider the olive tree, which in the Mediterranean is both omnipresent and widely used yet is known simply as the “olive tree.” On the other hand, some plants’ names are quite similar in the different languages of the places where they grow, for example, *Potentilla reptans* L*.* is called “five fingers herb” (or names containing the words “five,” “fingers,” “hand” or “foot”) in English, French, Spanish, Portuguese, German, Russian, Greek, Romanian, Polish, Lithuanian, Catalan, Basque, and Chinese, due to the leaf morphology [4]. In Serbo-Croatian, “petoprsnica,” literally meaning “of five fingers.”

A few studies have conducted a multilingual comparison of the same plant species to understand its performance, perception, and use across its area of distribution. Flattery and Swartz [5:141–152] study the identity of the mythical “Haoma” plant, which plays an important role in Zoroastrian worship. In addition to historical–geographic analyses, they employ a multilingual comparison of the common names of *Peganum harmala* L. (and other plant species suspected of being “Haoma”). Linguistic analysis is used by these authors as another tool to establish the plant’s identification. A similar approach is adopted by Šeškauskaitė and Gliwa [6] when studying the etymology of *Datura stramonium* L*.* and related narcotic species in Lithuania. Austin and Felger [7] study the etymology of the genus *Fagara* (Rutaceae), from its first written record in the eleventh century through to the present day. They employ historical, economic, geographic, linguistic (in several languages) and ethnobotanical approaches to understand the origin of the plant and its economic trade route. Austin [8] studies the history and etymology of *Sambucus* to reveal the history of the intercultural exchange of this plant and the evolution of its name. Dafni et al. [9] reconstruct the etymological, ethnobotanical, and folkloristic roots of 290 vernacular names of *Ecballium elaterium* (L.) A.Rich. in 38 languages. They use the plant’s morphological data, ecological characteristics, medicinal properties and uses, as well as historical evidence and ethnobotanical data, to explain the meaning, origin, spread, and history of the plant names.

Mandrake is perhaps the most famous medicinal plant in western culture since biblical times and throughout written history. This view has been clearly expressed by several authors, with statements such as: “Of all the medicinal herbs used in the ancient and medieval world, none was regarded with as much fear or wonder as the mandrake”; Silberman [10:89] noted that “Of all the plant illustrations (in medieval herbals) representing mythological beliefs, superstition, or witchcraft, the one that comes uppermost to mind is the mandragora or mandrake.”

Moreover, several well-known writers have devoted passages to the plant and its properties, from William Shakespeare’s many plays (see [11]), including *Othello*, *Macbeth*, and *Romeo and Juliet* (e.g., “And shrieks like mandrakes torn out of the earth” Act IV, Sc. 3), to Niccolo Machiavelli’s *La Mandragola*, the Nobel Prize-winner C. J. Cela’s *Diccionario del Erotismo*, and the recent and popular J. K. Rowling’s *Harry Potter and the Chamber of Secrets*.

### Aim of the study and hypotheses

Waniakowa’s [12] survey of the mandrake names is limited to linguistic aspects mostly of northern and central European languages and deals especially with the comparison of the common names with those of *Atropa belladonna* L*.* She relies extensively on ethnobotanical information to elucidate the meanings of these plant names in Europe. Most of this geographic area is beyond the mandrake’s natural distribution, i.e., mainly the Mediterranean area spreading eastward to Iran and the Caucasus.

In the present study, we cover a larger geographic area and more languages in search for mandrake names. We use the plant’s morphological data, ecological characteristics, medicinal properties and uses, as well as historical evidence and ethnobotanical data, to explain the meaning, origin, spread, and history of the plant’s names. The study was done to pinpoint the putative geographic origin and cultural distribution of the myths related to the mandrake based on the distribution and the origin of names throughout history. Our working assumption is that a proliferation of mandrake names in any specific language or category (see below) may reflect its ritual and/or practical importance and/or intimate knowledge related to this plant (see above; [1:1–2]). Based on our analysis, we examine four hypotheses:

*Hypothesis* *1* Due to the longer history and broader distribution around the Mediterranean (including the Middle and the Near East), one expects to find more cultural migrations of names (and myths and customs reflected in these names) from east to west (Middle East to Europe) and from south to north (southern Europe to eastern, central, and northern Europe) than migrations in the opposite directions.

*Hypothesis 2* One expects to find more plentiful names (and in more categories, see below) in countries in which the mandrake is native (especially around the Mediterranean) and has more ancient history than in other parts in Europe in which the mandrake (and its legends) arrived later. This would particularly relate to the appearance of the plant (e.g., morphology of aerial parts, root, fruits) and their similarity to other plants and animals including humans, and to its uses and effects.

*Hypothesis 3* Since the mandrake has a long history as an aphrodisiac and as an omnipotent medicinal plant over generations (see [13]), one expects to find relatively many names which are related to these categories.

*Hypothesis 4* Witchcraft and black magic were highly developed in Europe [14, 15], and involved hallucinogenic and narcotic plants [16:passim], while in the Muslim world, they are strictly condemned [17:1356, 18:passim, 19:passim]. Thus, one expects to find more names related to this category in Europe than in the Muslim world.

## Materials and methods

### Data gathering and possible pitfalls

Popular names for *Mandragora* spp. were collected in as many languages as possible through: literary reviews, dictionaries, the authors’ personal knowledge, and rarely in internet sources (where the plant’s identity was not doubtful). All the names were checked by us as well as by local botanists to ascertain their validity as well as their meaning in each language. Dubious or wrong names not clearly related to *Mandragora* were discarded.

We have focused on the names of the two species generally recognized as growing wild in the Mediterranean area: *M. officinarum* L. and *M. autumnalis* Bertol. (see [20–22] for nomenclature and taxonomy), excluding the Asian species usually considered as different ones *M. caulescens* C.B. Clarke and *M. turcomanica* Mizg. and their related synonyms. This approach was taken for several reasons: 1. The taxonomical distinction between M. *officinarum* L. and *M. autumnalis* Bertol. is debateable even among specialists [20–23]. We have not found any traces of distinction between the vernacular names of these two species. 2. A significant amount of European mandrake descriptions and iconography since antiquity but also in late medieval times and the Renaissance, refer to a distinction between male and female plants, known as *mas* (male) or *foemina* (female). While in antiquity, the distinction was made according to the root shape (thin for female and thick for male ones; e.g., Dioscorides, Pliny), some authors suggest that plants with ovoid fruits (more abundant in Central and Eastern Mediterranean, attributed to *M. officinarum*) were considered male mandrake, whereas plants with spherical fruits (more abundant in W & S Mediterranean and attributed to *M. autumnalis*) were considered female (e.g., [24, 25])*.* Therefore, this distinction seems to be more or less arbitrary through time. 3. The Iranian-Caucasian species (*M. turcomanica* Mizg.) shares, largely, the same medicinal uses as the former two species, which indicates a large-scale cultural migration between the Mediterranean and the Irano-Turanian species [13] as well as many rituals (see below). Secondary sources (e.g., [26–31]) were scrutinized as carefully as possible for the validity of the mandrake names by comparison to the original texts in each language and personal knowledge of the authors.

Considering the validity and reliability of the names used for mandrake in the various languages, we tried to look out for, and avoid, the following possible pitfalls:

In several countries in which the mandrake is not native (e.g., Northern, Central, and Eastern Europe), there is a tendency in the literature to adopt, transliterate and translate the mandrake’s names from other languages (especially from Ancient Greek and Latin as well as from the Arabic). Authors just translate/transliterate old literary names into their own languages, while these names were practically not used in the vernacular. This discrepancy is obvious when botanical texts are compared to folkloric/literary ones.

It is worth paying attention to distinguish between names that were given to the mandrake in its natural distribution in comparison to countries into which it was introduced (as a garden plant and as medicine, for ritual, and/or witchcraft purposes). It is assumed that as the folk tradition of the plant in these countries is historically shorter, some names in these languages may reflect locally evolved beliefs and traditions from the periods after the plant introduction.

Some of the reviewed literature used the name “mandrake” when the botanical identity is that of species of other genera such as: *Atropa* (rev. [12]), *Bryonia* (see [32:111, 33:193, 11:144], and *Podophyllum* [34, 35: 495]. Also, less frequently other species such as: *Datura stramonium* L*.*, *Valeriana officinalis* L., *Aconitum tauricum* Wulfen, *Gratiola officinalis* L., *Hyoscyamus niger* L., *Leonurus cardiaca* L*.*, Cicuta *virosa* L*.* and *Peganum harmala* L. [36: 3–4], *Chelidonium majus* L. [37: 273], *Allium victorialis* L. [38: 120–121] and *Tamus communi*s L. [39: 204] were named “mandrake.” Thus, it is not surprising that Eliade [40] in his famous article “The Cult of *Mandragora* in Romania” referred to *Atropa belladonna* as “mandrake” and not *Mandragora officinarum* (that was previously in the genus *Atropa*, which is not indigenous to Romania). As a result, since several authors cited Eliade’s work (e.g., [41–44]), *Atropa belladonna* was related to *Mandragora*.

In the literature, several times a name is given from a certain language as a proper name from a different language. For example, the mandrake names *anthropophora* and *xērà ánthē* are regarded as “Latin” names as in the consulted works (Dioscorides’ *Materia Medica* [45: IV,75]) are considered so, but these have been directly transcribed to Latin from Greek, and these names are probably better considered as just Greek names and were omitted from our analysis.

In the same sense, in some territories, a certain name for the plant may have arrived with migrants, sellers, or any citizen, or even from the medical literature together with medical texts. In these cases, names could be better interpreted as a cultural migration and not as a vernacular name originated in this specific territory. An example is the name *mandragora* in Catalan. Once it is explained that the origin of this name is more probably Greek than Latin, and since Catalan is a Romance language, it seems that the very first references recorded in Catalan in the thirteenth-to-fourteenth centuries (firstly: *mandràgola*; secondly: *madragora*; see [46]) come from a Catalan context but were originally published in an Arabic text, afterward translated to Catalan and later inserted in medical (official medieval medicine) texts. This first mention in Catalan is a translation of a previous work written in Arabic by the same author (Ramon Llull [46]), and translated by himself into Catalan (Llull used Latin, Arabic and Catalan alternately in his writings on the thirteenth century in an attempt to spread his “scientific” ideas through Mediterranean cultures). The reference to *mandràgola* is even earlier than *mandràgora* and comes from the first scientific texts written in Catalan.

### Data presentation

Spelling variants of the same name were pooled into the same cell in the tables and counted only once. To avoid repetition, we cited each author only once in the relevant cell even if he presents several spelling variants. This method had no influence on our conclusions. Sometimes, especially with respect to *alraune* in German, distinguishing between a “derivative” (i.e., derivatives from a generic name, as *mandragora* or *alraune*) and a “spelling variant” (e.g., *mandracola* instead of *mandragora* in Spanish; about sixty variations are scattered in the literature; see Table 1) is quite arbitrary. The same is the case with regard to transcriptional corruptions of Farsi and Arabic names in European languages (e.g., *abrusanam* and *yabroukh*). For language names and categorization, we followed the website ethnologue (https://www.ethnologue.com/).

The names were grouped, in the light of linguistic and ethnobotanical aspects, into the following categories: **A**. Derivatives of “generic” names (with their variants in transcription): 1. *Mandragora*; 2. *Alraune*; 3. *Yabroukh.* **B**. Morphological characteristics: 1. Root (Anthropomorphism); 2. Leaves, Fruits, and Seeds; 3. Similarity to other plants. **C**. Pharmacological characteristics and medicinal uses: 1. Medicinal properties; 2. Narcotic; Hallucinations; Poisonous; 3. Aphrodisiac. **D**. Magic and witchcraft: 1. Black magic—sorcery, witchcraft, and magic, Bad luck, Evil eye; 2. Evil supernatural agents, Satan, the devil, genie, monster, dragon. 3 White magic—Good luck, talisman, dolls. **E**. Pulling-out ceremonies: 1. Screaming, groaning and voices; 2. Use of a dog; 3. Shining and lights; **F**. Plant conception: Gallows and hanging: 1. Plant originated from human semen, urine; 2. Creation of Adam. **G:** Relationship to historical and mythological characters: 1 King Solomon, Circe, and Prometheus; 2 Elephant. **NC**: Not classified. There are other names whose meaning/origin is not clear, or are not related to any of the above-defined categories. A very few names (e.g*., Mandragora mannetje* [“Mandrake man”] and *pisduiveltje* [“Little piss devil”] in Dutch) may be classified into two categories. In these cases, we decided on the category according to relevance in the original text.

## Results and discussion

**Fig. 1:**
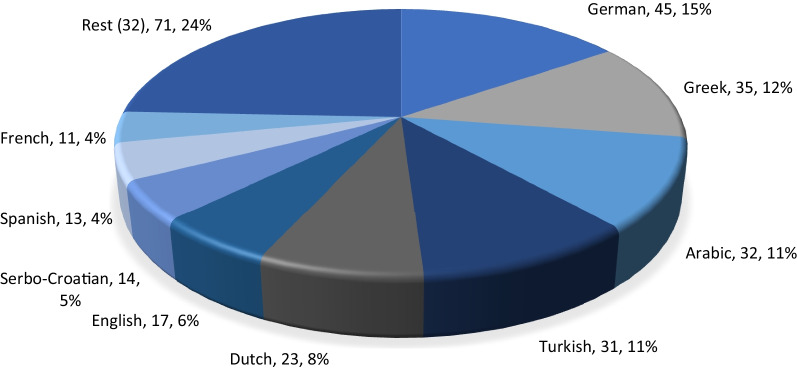
Languages which comprise more than ten vernacular names for mandrake; number of names and percentages

Our database with 292 names for the plant covered, as mentioned, a total of forty-one languages spoken from antiquity to the present day in the area where Mandragora officinarum (including M. autumnalis) is widely distributed and/or used. This includes two dead languages (Latin and Old German). There are nine languages for which more than ten vernacular names for mandrake were recorded (see Table 8; Fig. 1). It is somehow surprising that four of these nine languages are not from the territory in which the plant is native (German, Dutch, English, French), and that at least in one more (Serbo-Croatian) the plant has been considered extinct [47], while in Greece, Arabic countries of North Africa, Spain, and Turkey, it is currently wild [22]. These nine languages cover 74% of all names, while the remaining thirty-three languages represent 26% of names.

**Table 1:** A. Derivatives of “generic” names: A1. *Mandragora*; A2. *Alraune*; A3. *Yabroukh*

Language/sub-category	Name	Ethnic transcription	Meaning	Selected references
*A1*				
Albanian	*mandragora, madëronë, matergonë*			Albanian Bible [12:163]
Armenian	[*mandragora*]			[48: 1268]
Bulgarian	[*mandragora*]	maндpaгopa		[49: 36]; [50]; [51: 394]
Catalan	*mandràgola, mandràgora*			[46: 190] (13th c.)
Corsican	*tramangula*			[52: 471]
Dutch	*mandragers kruit*		[“mandrake herb”]	[53: 11]; [31: 45]
Dutch	*mandragore*			[31: 45]
English	*mandrake* Spelling variations: *mandrage,* *mandrag, mandragge,* *mendrage*			[54: 51]; [55: 322]; [56:66]; [28: 71]; [31: 43]; [30: 333]
French	*mandrigoula*			[28: 71]; [31: 44]
Georgian	[*mandragora*]			Rainer Bussmann (Pers. Comm 25.1.20)
Greek	[*mandragora*] Spelling variations: [*mandragúda, mandragúdas, matragúra, mantragúras,* *mandragúri****,*** *mandraúla,* *mandraúna*, *mentragúra*]	μαντραγόρα Spelling variations: μαντραγούδα, μανδραγούδας, ματραγούρα, μαντραγούρας, μαντραγούρι, μανδραούλα, μανδραούνα, μεντραγούρα		[57: 173]; [58: 2509]; [59: 433,437]; [60: 408]
Greek	[*mandragóras*] *(m)*	μανδραγόρας		[45: IV, 75]; [58: 2509]; [59: 440]; [61: 9,8,8]; [62: 600]
Hungarian	*nadraguyla*			[63: I, 537]; [64: 55]
Italian	*mandragora* Spelling variations*:* *mandræela, mandràhura, mandràgura, mandràura, mandulàgrona, mandràgura*			[65]; [28: 71]
Polish	*matryguna, matrygan, medregula*			[12: 170]
Portuguese	*mandragora, mandragola*			[66]
Russian	[*mandragora*]	Maндpaгopa		[67: 75–76]; [68: 411]; [69: 187]
Serbo-Croatian	[*mandragora*]	Maндpaгopa		[70: 291]; [71: 20]
Serbo-Croatian	[*mandragula*]	Maндpaгyлa	In Serbo-Croatian it is easier to pronounce “*mandragula*” than “*mandragora*”	[70: 291]
Serbo-Croatian	[*narangulina*]	Hapaнгyлинa	Probably a variation of “*mandragula*”	[71: 20]
Spanish	*mandrágora* Spelling variations: *mandrágora, mandracola, mandrácola*		Frequently adjectivized as “*mandragora macho/hembra*” (male or female) or “*mandragora de flor azul/blanca*” (regarding flower’s color)	[66]; [72: 585]
Spanish	*mandrágula,* *mandragula, mandrácula*			[66]; [72: 585]
Turkish	*mandırağa*		[“Landlord Mandir”] (folk etymology of mandrake)	[73:107]
Ukranian	[*mandrygula*]	aндpигyлa		[12:163]
*A2*				
Danish	*alrune*			[74:160]
Dutch	*alrüneken, alrunik*			[75: 229]; [76, I:95]
Dutch	*alruyne, alruin*			[77: 333]; [74: 160]; [28: 71]; [31: 45]
Finnish	*alruna*			[78:64]
German	*alraun* (+ ca 60 spelling variations; see text)			[79: 5]; [75: 229]; [28: 71]; [31: 41]; [30: 329]; [29: II: 336]; [27: 344]; [80: 19]; [81: 23] [82];
Norwegian	*alrune*			[74: 169]
Serbo-Croatian	[*alrauna, alrun*]	aлpayнa, aлpyн		[71: 20]
Swedish	*alruna*			[83: 258]; [74: 160]
Swedish	*alrunsrot*		[“*alrune* (mandrake) root”]	[63; I:23]
*A3*				
Arabic	[*ež-žarbūḥ*]	الجربوح		AGK Pers. obs. (Palestine)
Arabic	[*yabrūh*, *yabróâh*]	يبروح		[84: 351] (Andalusia, 6–7th c.); [85, II: 773] **(**Andalusia, 11th c.)
Arabic	[*yabrūh, yabrūx*]	يبروح ، يبروخ		[86: 207] (Arabia, 9th c.); [87: 54] (Arabia, 10–11th c.); [88: 592]; [89: 299] (Jordan); [90: 115] (Palestine); [91: 203] (Syria); [84: 351] (Andalusia, 6–7th c.); [85: II: 773] (Andalusia, 11th c.); [92: 26] (Morocco)
Aramaic	[*yabroukh, yabroukha, yabroukhin*]	יברוח, יברוחא, יברוחין	See text concerning the etymology of *yabroukh*	Onkelos (Rome, 1st c., Aramaic translation, Genesis 30:14 [93: 14]); Babylonian Talmud [94]
Bengali	[*yebruj*]			[28: 71]
Farsi	[*sāyeh-borūj*]	سایه بروج	Derivative of *yabroukh* in Aramaic (see text)	[95: 645]
Turkish	*Yebrûh*		See text concerning the etymology of *yabroukh*	[73: 107]; [41: 124]; [96: 232–234]
Turkish	*yebrûhu’s-sanem*		Idol’s *yabroukh*	[97: 1391]; [73: 107]; [41: 124]; [96: 232–234]

### Derivatives of “generic” names

*The origin of “mandragora”:* The origin of the Greek μανδραγόρας is obscure [41: 115, 91: 256, 149: 316]. The main suggestions to explain the origin of this name are as follows:

1. Persian origin from *mardum-giyah* (plant–man) [41: 115, 207: 237]. This idea is rejected by Asatrian [100] based on the arguments that this plant has no name in old Persian texts and because in the historical period it was already an extinct (or sporadic) species within the floristic nomenclature of Iran.
2. Greek origin: *‘mándra’* = “stable” and *‘agora’* = “gathering place” thus referring to the places where it was commonly found [208: 302, 120: 1835]; although this explanation was refuted by Genaust [208: 237].
3. Greek origin: *‘mandragoros’* is derived from ‘*mándra*’ = “an oxtail,” sometimes relating to cattle, and *‘aguros*’, cruel, on account of its poisonous effect on cattle [209, II:132, 210: 355].
4. Greek origin: may be a variation of the Greek *‘mandragoritis,’* an alternate name for Venus [79: 20, 211: 3, 122: 260, 176: 241]. This idea was refuted by Genaust [208: 237].
5. Sanskrit origin: a. *‘mand’* = *“*joy*,” “*intoxication*”* [91: 256].
6. Sanskrit origin: b. *‘mantasana’* = “sleep,” “life,” or *‘mandra’* = “pleasure” [91: 256].
7. Sanskrit origin: c. *‘mantara’* = “paradise tree” and *‘aryu’* = “unmarried, violently passionate” [91: 256].
8. Sanskrit origin: d. *‘mandros’* = “sleep” and *‘agora’* = “substance” [176: 241; 149: 316] (see also [212: 22–23]).

We found 24 names in 19 languages in which the plant is called mandragora and/or its derivatives.

Most of the variants were in Greek (8) and in Italian (6), and most of the derivatives are from European languages (except one in Turkish and one in Georgian). We consider this as indirect evidence for the European (Greek) origin of the derivatives of this name (Table 1), as some dictionaries reflect (e.g., [213]). Asatrian [100] also analyzed the Farsi names and concluded that the origin of the name *mandragora* is Greek and not Iranian.

**Table 2:** B. Morphological characteristics: B1. Root (anthropomorphism); B2. Leaves, fruits, and seeds; B3. Similarity to other plants

Language/sub-category	Name	Ethnic transcription	Meaning	Selected references
*B1*				
Arabic	[*šağarat aṣ-ṣanam*]	شجرة الصنم	Lit. [“The image’s (idol) tree,” “a human (shaped) tree”]	[88:14]; [98, IV: 443]; [99]
Armenian	[*marda-khot*]		Literarily [“Human (-like) plant”]	[100: 106]; [101: 152]; [102: 251]
Czech	*mužijk, mužicek*		[“little man”]	[103: 43]; [104: 289]; [12: 166]
Czech	*strýček*		[“uncle”]	[104: 289]
Danish	*dukkeurt*		[“doll’s herb”]	[27: 344]
Dutch	*aardmannetje*		[“little earth man”]	[76, I:95]; [31: 45]; [105: 35]
Dutch	*alruinmanntje*		[“mandrake’s little man”]	[106:29]
Dutch	*mandragora mannetje*		[“mandrake’s man”]	[28: 71]; [31: 45]
Dutch	*wortelmannetje*		[“little root man”]	[107: 63]; [31: 45]
Dutch	*wortelmensch*		[“root man”]	[108: 541]; [31: 45]
English	*ladylin*		[“little lady”]	**[**29, II: 336]; [109: 70]
English	*root of life*		Due to the hallucinogenic effects?	[30: 334]
English	*womandrake*			[110: 343]; [56: 66]; [109:70]; [29, II: 336]
Farsi	[*mardom-gīyāh*]	مردم گیاه	[“plant of the people”]	[100: 106]; [111: 2]: [101: 152]
French	*homme planté*		[“planted man”]	[28: 71]; [31: 43]; [29: 336]
French	*plante humaine*		[“human Plant”]	[112: 225]; [28: 71]; [31: 44]; [113: 8]
German	*alraunmännchen, alruyn manneken*		[“alrun man”]	[31: 41]; [114: 15]
German	*atzelmännchen*		[“doll”]	[115:164]; [30:330]
German	*atzmann*		[“doll,” “puppet”]	[31]; [30]; [114: 15] [81: 23]
German	*erdmännchen, erdmännlein*		[“little earth man”]/[“earth mannekin”]	[116: 25]; [30: 331]; [31: 41]; [114: 15]; [81: 23]
German	*erdweibchen*		[“little earth woman”]	[117: 185]; [116: 25]
German	*mandlwurz, mandelwurz*		[“little root-man”]	[118: 355]; [119: 137]; [120: 113]; [2: 1138]
German	*menschenwurzel*		[“human’s root”]	[114: 15]; [81: 23]
Greek	[*anthropómorphos*]	ἀνθρωπόμορφος	[“human-shaped”] (due to its anthropomorphic roots)	[45: IV,75]
Greek	[*paidí*]	παιδί	[“child”] (may be because of its small, child-shaped root; less probable is because it may induce fertility)	[59: 442]
Greek	[*arkánthropos*]	ἀρκάνθρωπος	[“bear-man shaped”] (due to its fat/hairy roots)	[62: 600]; [58: 2509]; [121: 357]
Latin	*antropophora*		[“human-bearer”] (from Greek)	[45: IV,75])
Latin	*semihomo*		[“half human”]	[45: IV, 75]
Polish	*męzyk*		[“male”]	[12: 164]
Turkish	*adamkökü*		[“man root”]	[122: 268]; [73: 107]; [90: 232–234]; [41: 124]
Turkish	*adamotu*		[“man plant”]	[123: 21]; [124: 2]
Turkish	*insan kökü*		[“person root”]	[73: 107]; [28: 71]; [93: 232–234]; [41: 124]
Turkish	*insan otu*		[“person plant”]	[73: 107]; [123: 21]; [124: 2]
*B2*				
Arabic	[*fākihat al—gurāb*]	فاكهة الغراب	[“raven’s fruit” [ (the birds like this fruit)	[125: 624–625] (Andalusia, 13th c.)
Arabic	[*sābizāj*, *ṣābizāj*]	صابيزاج,سابيزاج	[“a plant with black (dark) seeds”] from Farsi: “*šā(h)*” which means black (cf. *šāh-tūt*) and “*bīzak”* means seed, grain	[84: 351] (Andalusia 6–7th c.); [126: 219]; [100: 106] (Syria)
Arabic	[*luffāh*]	لفّاح	“The burning (or emitting a good odor) fruit” (name related only to the fruit of the plant)	[86: 107] (Arabia, 9th c.); [85, II: 774] (Andalusia, 11th c.); [88: 592]; [41: 121]; [127: 285]; [128: 242] (Arabia, 9th c.); [129: 250] (North Africa); [130: 36] (Turkey); AGK Pers. obs.(Palestine)
Armenian	[*loshtak, loštak*]		*Loshtak* means literally “ear” (due to the fact that “the leaf is large and with many ridges like an ear” (Garnik Asatrian, Pers. com. 30.10.19)	[63, I: 537]; [101:154]
Farsi	[*šā(h)bīzak* *Spelling variants: sābisaj / šbizak / sbysq, š’bysk*]	شابیزک	[“plant with black seeds”] (see above, also *A. belladonna*)	[130: 36]; [95: 688]; [100: 106]
Greek	[*avgoulátsa/ avgoudátsa*]	αυγουλάτσα, αυγουδάτσα	[“bearing little egg-shaped fruits”]	[59: 430]
Greek	[*chondrovotáni*]	χοντροβοτάνι	[“fat herb”] (probably due to its large leaves or its fat taproot)]	[59: 439] (Lakonia)
Greek	[*kourouniá*]	κουρουνιά	[“crow nest-shaped”] (leaves)	[59: 433–434] (Nisyros and Leros islands)
Greek	[*megalovotáni*]	μεγαλοβοτάνι	[“large herb”] (due to its large leaves or fat taproot)	[59: 439] (Lakonia)
Greek	[*papútsa*]	παπούτσα	[“shoe-shaped”] (leaves)	[59: 440] (Cyprus)
Serbo-Croatian	[*nadliška*]	Haдлишкa	“*Nad*” means over, above; “*liška*” means leaf. Probably the word means something stronger or more important than leaf (which is close to the root in the mandrake) or could also emphasize the fruit (“above the leaf”)	[71: 20]
Serbo-Croatian	[*veliko zelje*]	beликo зeљe	“*Veliko*” means great, large, big; “*zelje*” means greens or herb. The word could implicate “a great herb” because of its relatively large (long) leaves	[71: 20]
Serbo-Croatian	[*vodopić*]	boдoпић	“*Voda*” means water, “*piti*” means to drink; literally “*vodopić*” is one who drinks water; could be linked to shiny intense green leaves (?)	[71: 20]
Serbo-Croatian	[*veliko zelje* / *velje zelje*]	beликo зeљe	“*Veliko*” means great, large, big; “*zelje”* means greens or herb. The word could implicate “a great herb” because of relatively large (long) leaves	[71: 20]
Spanish	*lirios*		[“lily flower”] (resemblance to lily flower)	[131: 175]
Turkish	*beş damar otu*		[“five-veined plant”]	[41: 124] (North Cyprus)
Turkish	*lüffâh*		*Luffah:* mandrake’s fruit in Arabic	[41:124]; [87: 293,340]
Turkish	*lüffâh-ı berry*		[“wild luffah”] (see *Luffah*)	[41: 124]
*B3*				
Arabic	[*tuffāḥ al-barr*]	تفّاح البرّ	[“wild apple”]	[132]
Arabic	[*tuffāḥ bittanžān*]	تفّاح بطنجان	[“eggplant’s apple”]	A local name in the Galilee. Israel (SAH Pers. Obs)
French	*belladone sans* *tige*		[“belladonna without a stem”]	[31: 43]; [28: 71]; [29: 336]
French	*pomme terrestre*		[“earth’s apple”]	[112: 225]; [133: 184]; [113: 8]
German	*borchart*		*Burcher:* a popular name for *Atropa belladonna* (Hambel 2002:330)	[75: 229]; [134, III: 53]; [31: 41]; [30: 330]
German	*erdapfel, ertapfel*		[“Earth’s apple”]	[83; 258]; [118: 355]; [119: 137]; [75: 22]; [134, III: 53]; [31: 41]; [30: 331]
German	*malzapfel,* *maltzapfel,* *melzlh apfel*		Seems to be a corruption/translation of “*pomum macianum*”	[136, I:2021]; [135: 23]; [137: 84]; [134, III:53]; [81: 23]
Greek	[*mala silvestria*]	μάλα σιλβέστρια	[“wild apples”]	[45: IV,75] (Romans in Greece)
Greek	[*mala terrestria*]	μάλα τερρέστρια	[“Earth's apples”]	[45: IV,75]
Greek	[*milopeponiá*]	μηλοπηπονιά	[“apple-melon tasting”]	[62: 600]; [121: 357] (Cyprus); [59: 440]; [60: 408]
Greek	[*miliákos*]	μηλιάκος	[“apple-like”]	[60: 408] [58];
Greek	θριδακία (f.) / θριδακίας (m)	ɵριδακία (f.)/ɵριδακίας (m)	[“lettuce-looking plant”]	[45: IV,75]; [61: 9,8,8]
Latin	*malum terrae,* *malus terrae, mala terrestria*		[“Earth’s apple” / “Earth’s apples”]	[84: 351] (Andalusia, 6-7^th^ c.); [79: 6] (Spain). [45: IV,75]
Latin	*thridakía, thridaks*		[“lettuce-looking plant”]	[45: IV,75]; [138: 419]
Serbo-Croatian	[*divlja jabučica*]	Дивљa Jaбyчицa	[“wild small apples”] “*divlja*” means wild and “*jabučica*” means small apple	[71: 20]
Slovak	*Pěkná jablečka*		[“beautiful apple”]	[139: 359]
Spanish	*manzana de tierra*		[“Earth’s apple”]	[131: 192]
Spanish	*acelgón, acelgones*		[“chard”] (due to the leaves resembling this plant)	[66]
Spanish	*berengenilla, berenjenilla*		[“little eggplant”]	;[66] [72: 585]
Spanish	*berenjena mora*		[“Moorish eggplant”]	[66]
Spanish	*lechuguilla*		[“small lettuce”]	[66]
Spanish	*tomatico*		[“small tomato”]	[66]
Spanish	*uva de moro*		[“Moorish grape”]	;[66] [72: 585]
Turkish	*lüffâh-ı berri*		[“Earth loofah”]	[41: 124]
Turkish	*toskafa kavunu*		[“butting head melon”] (because it looks like a head that butts)	[73: 107]; [123]
Turkish	*yer elması*		[“Earth apple”]	[73: 73]; [123: 21]
Turkish	*yer yenidünyası*		[“Earth’s loquat”]	[41: 124]; [123: 21]

**Table 3:** C. Pharmacological characteristics and medicinal uses: C1. Medicinal properties; C2. Narcotic, hallucinogenic, poisonous; C3. Aphrodisiac

Language/subcategory	Name	Ethnic transcription	Meaning	Selected references
*C1*				
Arabic	[*abu* salām]	أبو سلام	[“father of health”]	[145: 709]
Arabic	[*ʾabd es-salām*]	عبد السلام	[“the servant of health”]	[122: 268]
Armenian	[*t ‘agawor amen* *Xotic*]		[“king of all (every) grasses (and forbs)”]; see text	[141: 389]; [101: 153]
German, Old	*ârzat uvûrze, arzanwurt, arzatwurz*		[“doctor’s root”]	[75: 229]; [137: 93]; [134, III:53]; [31: 41]; [114: 330]; [30: 330]; [142: 38]
German	*heilmännchen,* *heilmännlein*		[“healing male”]/healing mannikin	[120: 113]; [31: 41]; [81: 23]
Greek	[*fistulóriza*]	φιστουλόριζα	[“fistula-healing root”]	[143: 331]; [59: 427,435]
Greek	[*fistulóchorto*]	φιστουλόχορτο	[“fistula-healing herb”]	[59: 445]
Serbo-Croatian	*bunovina*	Бyнoвинa	“*bunilo*” (root of the word) means delirium, madness	[71: 20]; [70: 291]
Turkish	*ebîselâm*		[“father of health”]	[41: 124]; [124: 2]
Turkish	*hastalık otu*		[“sickness plant”]	[41: 124] (North Cyprus)
Turkish	*Kankurutan*		[“blood dryer”] (because it is believed that it stops bleeding; see text)	[144: 334]; [73: 107]; [28: 71]; [41: 124]; [123: 21]
*C2*				
Dutch	*doodkruid*		[“death's herb”]	[83: 258]; [28: 71]
Dutch	*slaapappel*		[“sleep’s apple”]	[145: 263]; [146: 940]
Dutch	*slaapkruid*		[“sleep’s herb”]	[146: 940]
English	*brain thief*			[109: 70]; [147: 169]; [148: 3]
English	*divine root*		May be due to its hallucinogenic effect	[30: 331]
English	*fool’s apple*		May be due to its hallucinogenic effect	[149: 316]
Gaelic	*codalian*		[“sleep apple”]	[83: 258]; [150: 139]
German	*doilwurz* Spelling variations: *doilworz dolwortz, dollwurz, tollwurtz, dilwurz*		[“mad root”]	[75: 229]; [134, III:53]; [120: 113]; [31: 41]; [30: 331]; [114: 15]
German	*dollblume*		[“mad flower”]	[81: 23]
German	*schlafbeere, schlafbeer*		[“sleep berry”]	[30: 334]; [81: 23]; [31: 42]
German	*tollkraut südliches*		[“southern mad herb”]	[119: 137]; [31: 42]
German	*schlafapfel,* *schlaf-aepffel*		[“sleep apple”]	[150: 693]; [119: 137]; [135: 23]; [134, III:53]; [31: 42]; [30: 334]; [81: 23]
German, Old	*twalm, tuualm*		[“sleep”]	[134, III:53]; [31: 42]
Greek	[*alítis*]	ἀλοῖτις	[“deceiving, alluring, seductive”]	[45: IV,75]
Greek	[*ippóflomos*]	ιππόφλωμος	[“driving horses dizzy”]	[59: 446]
Greek	[*mórion*]	μώριον	[“stupefying plant”]	[45: IV,75]; [61: 9,8,8]
Greek	[*trellóchortaro*]	τρελλοχόρταρο	[“madness herb”]	[59: 439]
Greek	[*vomvóchylos*]	βομβόχυλος	[“humming-inducing juice”; “intoxication-inducing juice”]	[45: IV,75]
Greek	[*rigaléos*]	ῥιγαλέος	[“shiver-inducing, thrilling”]	[45: IV,75]
Hungarian	*bolondfű*		[“fool's grass”]	[152: 41]
Spanish	*cerezas de sapo*		[“toad cherries”], maybe due to the poisonous effect of toads	[66]
*C3*				
Arabic	[*ˁarūsaḷḷa*]	**عروسالّة**	Diminutive of [“a bride”]	[125: 624–625] (Andalusia, 13th c.)
Arabic	[*ḥabb at-taˀlīf*]	حب التأليف	[“the fruit that gets the lovers close”]	[125: 624–625] (Andalusia, 13th c.)
Arabic	[*luˁba muṭallaqa*]	لعبة مطلقة	[“the expelled bride”]	[99]
Arabic	[*luˁba mu'allaqa*]	لعبة معلقة	[“the dependent bride”] (lit. “The married bride”)	[99]
Danish	*kærlighedsæble*		[“love apple”]	[153: 81]
Farsi	[*mehr gīyah, mihrgiāh*]	مهر گیاه	[“plant of love”]; “*meher*” means affection, “*gîah*” means grass	[154: 6 note 4]; [95: 152]
Finnish	*lemmenmarja*		[“love berry”]	[155: 261]
German (old)	*chindelina wurz*		[“little child’s root”] (see text)	[156: 118]; [31: 41]
German	*kindleinkraut*		[“child herb”]	[120: 113]; [114: 15]; [81: 23]
German	*liebesapfel*		[“love apple”]	[114: 15]; [81: 23]
German	*liebeswurz, liebeswurzel*		[“love herb”]	[114: 15]; [81: 23]
Greek	[*sernikobótano*]	σερνικοβότανο	[“male-birth-inducing herb”]	[59: 441]
Hebrew	[*duda'eem*]	דודאים	Probably related to "דוד" meaning love	Genesis 30:14–16; Song of Songs 7:13
Turkish	*ayıkotu*		“vivacity grass”	[157]: 128
Turkish	*muhabbet otu*		[“love plant”]	[73: 107]; [124: 2]; [41: 124]; [90: 232–234]
Turkish	*sevgi otu*		[“love plant”]	[41: 124]; [90: 232–234]
Ukrainian	[*lubovyća*]	любoвицa	[“love plant”]	[12: 162]

**Table 4:** D. Magic and witchcraft: D1. Black magic, sorcery, witchcraft; D2. Evil supernatural agents—Satan, devil, genie, monster, dragon; D3. White magic, good luck, talisman, dolls

Language/subcategory		Name		Ethnic transcription	Meaning	Selected references
*D1*						
Dutch		*toverwortel, tooverwortel*			[“magic root”]	[146: 940]; [158: 255]; [159: 333]; [28: 71]; [105: 35]
Dutch		*heksenkruid*			[“witches’ herb”**]**	[146: 940]; [105: 35]
Dutch		*heksen loverwortel*			[“witches’ love root”]	[28: 71]; [31: 45]
English		sorcerer’s root				[12: 166]; [109: 70]
English		enchanter’s nightshade				[54: 354]
English		witches’ herb				[149: 316]
Estonian		*nöiajuua*			[“magic root”]	Dainius Razauskas (Pers. Com.12.3.19)
French		*herbe aux, magicien, herbe des magiciens*			[“magician’s plant”]	[160: 91]; [28: 71]; [31: 43]; [29: 336]
German		*hexenkraut*			[“witches’ herb”]	[83: 258]; [119: 137]; [28: 71]
German		*zauberwurzel, zauberwurz*			[“magic root”]	[75: 229]; [134, III: 53]; [31: 42]; [30: 335]; [114: 15]; [28: 71]; [80: 19]
Hungarian		*varázsgyökér*			[“magic/miracle root”]	[161: 151]
Serbo-Croatian		[*skocelj* / *skočac*]		Cкoцeљ / Cкoчaц	The word “*skakati*” means jump, leap. Probably related to beliefs in a very “alive,” restless, and troublous root, especially when pulled out from the soil / of “*skocelj*”; “*skočac*” means one who jumps	[71: 20]
Russian		[*koldunova trava*]		кoльдyнoвa тpaвa	[“sorcerer’s herb”]	[83: 259]; [162: 1129]
*D2*						
Arabic		[*bēḍ el-ġūl*]		بيض الغول	[“monster’s eggs”]	[163: 34] (North Africa); [129: 167]; [92: 26]; AGK pers. obs. (Palestine)
Arabic		[*beiḍ el- ğinn*]		بيض الجنّ	[“genie’s eggs”]	[164: 114]; [165, II: 261] (Palestine)
Arabic		[*bayḍ al- ġūl, bayḍ el- ġūl*]		بيض الغول	[“Goula’s (witches’) eggs”]	[166: 47] (Morocco)
Arabic		[*xawx el- ğinn*]		خوخ الجنّ	[“the Jin's Peach”]	[167] (Lebanon)
Arabic		[*luffāḥ el- ğinn*]		لفّاح الجنّ	[“the burning (*or* emitting a good odor) fruit of the Genie”]	[129: 167]; [92: 26] (North Africa)
Arabic		[*tuffāḥ il-mağal*]		تفّاح المَجَل	Probably derivative of *Tuffāḥ il Mağann*	[168, I: 250] (Palestine, Gaza area)
Arabic		[*tuffāḥ il- ġūla*]		تفّاح الغولة	[“Goula’s apple”]	[163: 34] (Morocco)
Arabic		[*tuffāḥ al- ğinn*, Spelling variants: *tuffāḥ al-mağânîn, tuffāḥ al-mağan,* *tuffāḥat al -ğinn*]		تفَاح الجنَ تفَاح المجانين! / تفّاح المجن/ تفّاحة الجنّ	[“apple of the Genie/ apple of the genies”]	[84: 351] (Andalusia, 6–7th c.); [87: 54] (Arabia, 10–11th c.); [169, II: 21]; [170: 577] (Palestine); [164: 114]; [171: 87]; [172: 248] (Moorish Spain); [41: 121]; [89: 299] (Palestine); [90: 115]; [173: 73] (Palestine)
Arabic		[*tuffāḥ eš-šayṭān*]		تفّاح الشيطان	[“apple of Satan”]	[174: 587] (Palestine); AGK Pers. Obs. (Palestine)
Czech		*divelsappl*			[“devil’s apple”]	[29: 346]
Dutch		*duivelsplant*			[“devil’s plant”]	[29: 346]
Dutch		*appeldragend*			[“dragon’s apple”]	]^83^: 258]; [28: 71]
Dutch		*duivelsete*			[“devil’s food”]	[29: 3346]
English		devil’s apple				[175: 209]; [159: 332]; [176: 24]; [179: 64]
English		devil’s food				[180: 60]; [181: 85]
English		dragon’s doll				[149: 316]; [109: 70]
English		Satan’s testicle				[149: 316]
German		*drachenpuppe*			[“dragon’s puppet”]	[30: 331]; [114: 15]; [80: 19]; [81: 23]
German		*satansapfel*			[“satan’s apple”]	[151: 693]; [81: 23]
German		*teufelsapfel*			[“devil’s apple”]	[134, III: 53]; [28]; [31: 42]; [30: 335]
German		*unholdwurz*			[“demon’s root”]	[134, III: 53]; [28: 71]; [31: 42]; [30: 335]
German		*unholdkraut*			[“demon’s herb”]	[75: 229]; [28: 71]
German		*wichtelmännchen*			[“Imp”]	[120: 113]; [31: 42]; [30: 337]
Greek		[*kalánthropos*]		καλάνθρωπος	[“Goblin”] because their root looks like *Kalikántzaros*, the goblin in Greek folklore	[177]
Hungarian		*ördögalma*			[“devil’s apple”]	[178]
Polish		*czartawa*			[“the demon flower”]	[12: 164]
Turkish		*cinelması*			[“ghost’s apple”]	[73: 107]; [41: 124]
Turkish		*şeytan şalgamı*			[“Satan’s turnip”]	[41: 12]
*D3*						
French		*plante qui chante*			[“singing plant”] (see text)	[28: 71]
German		*geldmännlein,* *geldmännchen*			[“little money-man”]/[“money manikin”]	[83: 258]; [114: 15]**;** [29, II: 343]
German		*glücksmännlein,* *glücksmännchen*			[“little fortune-man”]/[“good luck manikin”]	[182: 88]; [120: 113]; [29, II: 343]
German		*hausväterchen*			[“little house father”]	[114]; [27: 344]; [81: 23]
German		*hinzelmannchen*			[“gnome”]	[118: 355]; [119: 137]

**Table 5:** E. Pulling-out ceremonies: E1. Screaming, groaning, and voices; E2. Use of a dog; E3. Shining and lights

Language/subcategory	Name	Ethnic transcription	Meaning	Selected references
E1				
French	plante qui crie		[“the screaming plant”]	[28: 71]
Polish	krzykaiec, krykwa		[“screamer”]/[“female screamer”]	[12: 164]
Polish	pokrzyk, pokrzyk białgłwi, pokrzyk samiec		[“the screamer”] / [“the female’s scream” or “the screaming female”] / [“the male’s scream” or “the screaming male”]	[12: 166]; [182: 180]
Polish	pokrzyk ziele, pokrzykowe ziele		[“the screamer herb”]	[83: 259]; [162: 1129]; [12: 164]
Russian	[pevenka trava]	пeвeнькa тpaвa	[“the screaming herb”]	[63, I: 23]; [27: 19]
Serbo-Croatian	[pokrik]	Пoкpик	The root of the word is “krik,” meaning scream or cry (see text)	[70: 291]
Turkish	hüngürük kökü		[“Sobbing root”]; it is believed that it sobs when pulled out from the earth	[73: 107]; [28: 71]; [41: 122,124]; [124: 2]
E2				
Arabic	[qātil al-kalb]	قاتل الكلب	[“dog’s killer”]	[125: 624–625] (Andalusia, 13th c.)
Dagestani (Avar)	xIapuleb xer		[“barking grass” or “grass (causing) barking”]	[184: 1486] cited by [102: 250]
Dutch	hondsappel, hundappel, hunderapfel		[“dog’s apple”]	[151: 42]; [28: 71]; [31: 45]; [102: 35]
Farsi	[sag-kanak]	سگ کنک	[“dog uprooter”] (= dog killer)	[95: 691]; [185: 200]; [186, III: 366]
Farsi	[sag-kuš]	سگ کش	[“dog killer”] (= dog slayer)	[100: 106]; [101: 152]
Farsi	[sag-šikan, sag-shekan]	سگ شکن	[“dog breaker”]	[100: 106]; [101: 152]
French	pomme de chien		[“dog’s apple”]	[112: 225]; [28: 71]; [3: 44]; [29: 346]; [113: 8]
German	hunds apfel, hunds apfelwurzel		[“dog’s apple”]	[83: 258]; [118: 335]; [135: 23]; [119: 137]; [134: 53]; [28: 71]; [114: 15]; [81: 23]
Greek	[mala canina]	μάλα κανίνα	[“dog’s apple”]	[45: IV,75]
Italian	mela canina		[“dog’s apple”]	[65] (Tuscany); [28: 71]
Italian	poma di cane		[“dog’s apple”]	[133: 184]; [77: 333]
Italian	mala canina		[“dog’s apple”]	[133: 184]
Turkish	köpek elması		[“dog’s apple”]	[73: 107]; [187: 15]; [188: 23]
Turkish	köpek otu		[“dog’s plant”]	[73: 107]; [187: 15–17]; [188: 23]
Turkish	köpektaşağı		[“dog’s testicle”]	[41: 124]; [123: 21]
E3				
Arabic	[al -yabrūḥ al-waqqād]	اليبروح الوقّاد	[“the burning mandrake”]	[132: 14]
Arabic	[sirāğ al-quṭrub]	سراج القطرب	[“devil’s candle” or “firefly candle”] (see text)	[87: 54] (Arabia, 10-11th c.); [122: 3]; [138: 246] (Andalusia, 13th c.); [88: 14]; [187, I: 49]; [190: 250]; [191, 891; No.246]; [28: 71]; [41: 121]
Russian	chortovaja svecha	чëpтoвaя cвeчa	[“devil’s candle”]; the Avarians believe that the mandragora emits light at night	[192: 143]

**Table 6:** F. Gallows and hanging: F1. Plant originated from human semen, urine under gallows; F2. Creation of Adam

Language/subcategory		Name	Ethnic transcription	Meaning	Selected references
*F1*					
Dutch		*galgejong, galgenjong*		[“gallows’ youngling”]	[31: 45]; [28: 71]; [102: 35]
Dutch		*galgenmann, galgemannekens, galgenmanntje*		[“gallows’ man”]	[107: 63]; [102: 35]
Dutch		*pisdiefje*		[“piss thief”]	[159: 333]; [31: 45]
Dutch		*pisduiveltje*		[“little piss devil”]	[159: 333]; [31: 45]
French		*madaglorie* Spelling variations: *magloire, madagloire, main-de-gloire, main degloire, maindeglorie, mandagloire, mandegloire, main de gore*		[“hand of glory”] (see text)	[56: 66]; [28: 71]; [31: 43]; [30: 332]; [29: 346]
German		*armesünderblume*		[“poor sinner’s flower”]	[30: 330]; [80: 19]; [81: 23]
German		*folterknechtswurzel*		[“torturer’s root”]	[30: 331]; [114: 15]; [80: 19]; [81: 23]
German		*folterwurzel*		[“torturer’s herb”]	[27: 344]
German		*galgenwurz*		[“gallows’ root”]	[81: 23]
German		*galgenmännlein,* *galgenmännchen*		[“little gallows man”] / [“gallows’ mannikin"]	[83: 258]; [119: 137]; [193: passim]; [134: 53]; [114; 15]
German		*henkerswurz, henkerswurzel*		[“executioner’s Root”]	[114]; [27]; [81]
German		*pissdiebchen, pissedieb*		[“piss thief”]	[119: 137]; [75: 229]; [134, III: 53]; [28: 71]; [31: 42]; [30: 334]; [81: 32]
Icelandic		*thjófarót*		[“thief’s root”]	[194: 44]; [195: 60]
Italian		*mano di gloria*		[“hand of glory”]	[65] (Sicily)
*F2*					
Russian		[*adamova golova*]	aдaмoвa глaвa	[“Adam’s head”]	[63: I: 1458]; [162: 1129]; [196: 335]; [197: 203]
Serbo-Croatian		[*Adamova glava*]	aдaмoвa глaвa	[“Adam’s head”]; “*glava*” means head	[70: 291]
Turkish		âdemotu		[“Adam’s plant”]	[73: 107]; [123: 21]

**Table 7:** G: G1. Relation to historical and mythological characters: King Solomon, Circe, and Prometheus; G2. Elephant

Language		Name		Ethnic transcription	Meaning	Selected references
*G1*						
Arabic		[*šajarat Suleimān]*		شجرة سلیمان	[“(King) Solomon’s tree”]	[99]
Armenian		[*Sołomon-* *imastunicaṙ*]			[“Solomon’s tree”] literally	[198: 97] (15th c.); [100: 106]; [101: 152]
English		Herb of Circe				[199: 224]; [29, II: 336]; [109: 70]
English		Herb of Prometheus				[200: 496]; [201: 121]
French		*circée*			[“Circe”]	[29: 336]
German		prometheuskraut			[“Prometheus herb]	[31: 42]
German		*zauberpflanze der circe*			[“Circe’s magic plant]	[28: 71]; [31: 42]
Greek		*kirkéa* Speliing variants: * kirkéon,* *dirkéa*		Κιρκαῖον / Κιρκαῖα / Διρκαῖα	[“Circe”]	[45: IV,75]
*G2*						
English		Elephant ear			See text	[202: 249]

**Table 8:** Non-classified names

Language	Name	Ethnic transcription	Meaning	Selected references
Arabic	[*labbāḥ*]	لبّاح (a variant of *Luffāḥ*)	[“makes a man brave”] (A hint for a potent man?)	[86: 107] (Arabia, 9th c.); [85, II: 449,774] (Andalusia, 11th c.)
Arabic	[*maġd*]			[86: 107] (Arabia, 9th c.); [85, II: 774] (Andalusia, 11th c.); [140: 1219]
Arabic	[*šuğğāˁ*]	شُجّاع	[“brave”] (A hint for a potent man?)	[168, I: 250]; [164: 114] (Palestine)
Basque	*urrillo, urrilo,* *urriloa,urriola*			[66]
Berber	[*tāryāl, taralya*]			[85, II: 774] (Andalusia, 9-11th c.); [203: 213]; [204: 257](Morocco)
Berber	[*ḥabb alʔilb*, *ḥabb attaʔlīf*]		“wild” seeds	[85, II: 774] (Andalusia, 9-11th c.)
Catalan	*albalarosa*			[84: 351]
Chinese	[茄参属]	qie shen shu	*Qie* 茄 in Chinese is Solanaceae (refers to plants in this family), 参 is suggesting a fat root or stem underground, like that of Ginseng	[205]
Greek	[diámorfos]	διάμορφος	[“double-formed; endued with various forms”]	[45: IV,75]
Greek	[*emionás*]	ἡμιονάς	[“mule’s plant”] (may be due to the use of mules to eradicate the plant?)	[45: IV,75]
Greek	[*kalánthropos*]/ [*kalanthropáki*]	καλάνθρωπος/ Καλανθρωπάκι	[“good man”] (euphemistic name) / [diminutive for “*kalánthropos”*]	[121: 357] (Cyprus); [62: 600]; [58]; [206: 78–79]; [59: 429]
Greek	[*kalanthropári*]/ [*kalanthropárin*]	καλανθρωπάρι / Καλανθρωπάριν	[“good-man shaped”] (euphemistic name); [diminutive for “*kalánthropos”*]/ [“good -man (shaped)”] (euphemistic name); [diminutive for *“kalánthropos”*]	[121: 357] (Cyprus); [206: 78–79] (Cyprus); [62: 600]; [58: 509] (Greece)
Greek	[*kaláthreptos*]	καλάθρεπτος	[“well-fed”] name probably based on the plant’s fat roots (see also the name *arkánthropos* above)	[206: 78–79]
Greek	[*kaláthrepos*]	καλάθρεπος	Corrupted from *“kalánthropos”* or *“kaláthreptos”*	[59: 436]
Greek	[*skalánthropos*]	σκαλάνθρωπος	[“good man”; “wooden man”]	[59: 431]
Greek	[*tátoulas*]	τάτουλας	Besides mandrake, also *Datura stramonium, Solanum nigrum* and *Atropa belladonna.* Seems to be a corrupted form of “Datura.”	[59: 431]
Latin	*aperium*			[45: IV,75]
Polish	*nasik*		May be related to the seeds?	[12: 164]
Serbo-Croatian	[*dliskva*]	Длиcквa	Word without meaning; “*liska*” means leaf; probably “*d*” as “*do*” meaning near; thus the word could refer to the importance of the part near to the leaf, i.e., the root, since mandrake is stemless, or the importance of a fruit	[71: 20]
Spanish	*vilanera, vinanera*		[“vinegar-taste plant”]	[66]; [72: 585]
Spanish	*vinagrera*		[“vinegar-taste plant”]	[66]
Syriac (Eastern Aramaic)	*bnat ganē*		name for the mandrake’s fruit	[186, III: 193]
Turkish	*at elması*		[“horse’s apple”]	[73: 107]; [123: 21]
Turkish	*bendavleo*			[41: 124] (North Cyprus)
Turkish	*hacılar otu*		[“pilgrim’s plant”]	[123: 21]; [28: 71]
Turkish	*hacı otu*		[“pilgrim’s plant”]	[73: 107]

*The origin of “alraun”*: Mandrake in modern German is known as ‘der Alraun’ or ‘die Alraune,’ which indicates the idea of a male and a female of the plant by referring to the shape of the root (two roots: female; more than two roots: male [214: 34]). The first occurrence of the plant name occurs in a 10th c. gloss of the biblical *dudaim* or else mandragora [30: 21, 142: 327]. The name in Old High German is *‘al-rūna,’* which transformed to Middle High German *‘al-rûne,’* which then became the Early and Modern High German *Alraun(e)* [215]. The German name is composed of the prefix *‘al-’* and the stem *‘rune’*. All etymological explanations of the name are highly speculative [214: 34]. There are several theories concerning its origin:

1. The name might be associated with the seeress Albrûna (Lat. *Aurinia*), who lived during the first century and is documented in Tacitus’s *Germania* [30: 22]; the theory is labeled highly unlikely [214: 34]. This theory can be found in Grimm [216, I:404–5], who relates that the names *alrūne/alrūn* (and the Nordic Aelfrûn, [214: 34]), to be identified with a wise-woman of the (Teutonic) antiquity from its old sense of a prophetic and diabolic spirit, has at length passed into that of the root (mandragora).
2. Etymologically, the word-stem ‘-rune’ can denote “to murmur, to whisper magic words,” or “whisper secrets, talk, spell” [30: 21, 214: 34].
3. The prefix *‘al*-*’* can either be related to the historic forms of *‘Alb’* (‘incubus’) or *‘all*, *groß’* (‘all, mighty’).

Combinations of these word parts suggest that the name means either “whispers/secrets of incubi,” or “knowing all magic,” or “mighty secret” [208: 237, 214: 34]. Referring to the highly uncertain etymology of the name, Kluge [214: 34]) also but hesitantly suggests the meanings “highly cleft” or “covered with wrinkles” as possible etymological interpretations.

We found nine names in seven languages in which the plant is called *alraune* and/or its derivatives. Most of these occur in different German variants (32); two variants are in Serbo-Croatian and the rest are Scandinavian languages.

*The origin of “yabrouh”*: The biblical (Genesis 30:14–16; Songs of Songs 7:14) “דודאים")[*duda'eem*]) was translated into Aramaic by Onkelos (35–120 BCE [93]) as “ יברוח” ([*yabrouḥ*]) and this name also appears in the Midrash, Bereshit Rabbah 72.5 (בראשית רבה [217]), a religious text from Judaism’s classical period, 3–5th c.) and the Talmud (4–5th c. BCE, Sanhedrin 99b; Erubin 10:26a [93]), as one of the names of the mandrake. This interpretation is accepted by the common Aramaic dictionaries (e.g., [218: 562, 219: 234, 220: 329, 221:197]) as well by the most important Jewish Bible commentaries (e.g., Avraham Ben Meir Ezra (Ibn Ezra), 12th c. [222] and Mōšeh ben-Nāḥmān = Ramban (Nachmanides), 13rd c. [223]). Although some other identifications for *duda’eem* have been suggested, e.g., by Rashi (Shlomo Yitzchaki 1040 –1105) in his authoritative interpretation of Genesis, they are not accepted by later authors (e.g., [224: 323–325, 186, III: 363–368]).

While there is general acceptance concerning the botanical identity of the *“yabrouḥ”* as mandrake, there are several debatable interpretations concerning the etymology of this name, as follows:

1. *Yavruḥa יברוחא*)), “the chaser” (the root b-r-ḥ means to chase); a plant which chases the demon away [225: 298 Note 189].
2. According to Wetzstein [226: 441] [*jebrûaḥ*] means “it (only) needs life.” This is equivalent to saying that the root is so like a human body that it only needs to have a soul breathed into it to become a small human being. The author relates this name to the Farsi [*medumgiâ*] = “the plant man.”
3. Ascherson [191: 890] mentions that today’s name for mandrake in Syria is [*ĝerâbûḥ*], which is likely an intentional corruption of the literary Arabic name [*jabrùḥ*], which would not provide the Syrians with a suitable meaning, whereas *bûḥ*, the final syllable of [*ĝerâbûḥ*], denotes sex drive, and thus the word can be given the meaning of “aphrodisiac,” as indeed the mandrake fruits were considered to be (in Genesis 30, 14) and still are today.
4. “Soul-giver,” “spirit-giver” *yavee* [יביא = “will bring,” *ruh* = [רוח] = “soul” [191: 890]. This author thinks that *yabrough* is Syro-Arabic. According to Elliot [227: 203], Aramaic *yahb-rouh* = giver of life.
5. Nathan Ben Yeshaya (Yemen 14thc.) explains: “*yabroukh*, since its nature is that the one who pulls it out will lose his spirit” [228:151].
6. According to Ibn Sina, *“*"*jabrol”* (a derivative of *yabroukh*) is the root of the mandrake and could be a name for any natural object, for instance a growing plant, in human shape (Ibn Sina, Canon of Medicine, Lib. II, Trsacii, Cap 365; cited by [229: 70].
7. Fleisher and Fleisher [230: 245] suggest that [*yabruĥ*] comes from Arabic [yâ-abu-er-ruĥ], which means “O master of breath,” (but in Aramaic it is the opposite meaning; see [231: 169].

We found eight names in five languages in which the plant is called [*yabroukh*] and/or its derivatives. Its derivatives appear only in Arabic (6), Aramaic (3), and Bengali (1). The Farsi name [*sāyeh-borūj*] may also be related to this group.

### Names related to the plant’s morphological characteristics

**Fig. 2:**
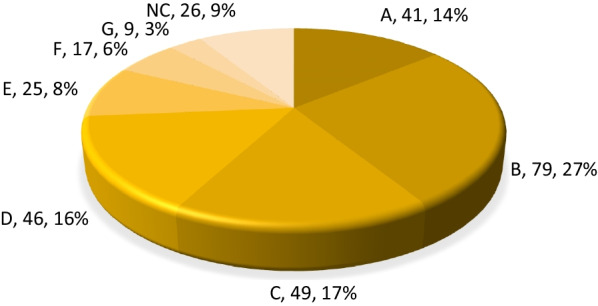
Distribution of names in main categories. Abbreviations for categories and subcategories as in Tables 1, 2, 3, 4, 5, 6, 7 and 8

Names related to morphological characteristics are the most frequent ones in all languages (up to 79 names within this category, 27% of total names, see Fig. 2), especially those connected with the root form and the similarity with other plants.

#### Root

Mandrake is a classic exemplar of the doctrine of signatures: this could be stated as form recapitulating function—physical characteristics of plants reveal their therapeutic value [232: 256]. The root resembles the human form (anthropomorphic)—symbolically, it is equivalent to the “root” of the human being, sexuality, and further, to fertility [11: 144] and love philtres [231, II:715]. Schultes and Hoffmann [117: 185] suggest that the European fear of the mandrake during the Middle Ages was due to doctrine of signatures. It was Pythagoras (in Dioscorides *Materia Medica* I.570 [45, I.570]) who gave the name *anthropomorphos* (ανθρωπομορφος, which means “shaped like a man”) to the mandrake. It is not surprising that 33 names in 13 languages are connected to the similarity of the mandrake’s root to the human body, of which seven are in German, six in Dutch, and four in French. Thus, the anthropomorphic view of the mandrake, as reflected in its names, appears especially in western European languages.

#### Leaves, fruits, and seeds

Few names are related to the shape of the leaves of the mandrake such as: *loshtak* [“ear”] in Armenian, [“five-veined plant”] in Turkish, and *papútsa* [“shoe-shaped leaves”] in Greek. These names are also related to other plant species. The Arabic name [š’bysk] originated from Farsi *šā(h)-* “black” (cf. šāh-tūt) and bīzak “seed, grain,” meaning “(a plant with) black (dark) seeds.” Isidore of Seville (Archbishop of Seville, c. 560–636 CE) wrote [84: 351]: “Mandrake is so called because it has a sweet-smelling fruit the size of a Matian apple; hence Latin speakers call it “apple of the earth.” Eighteen names in seven languages denote the resemblance of the mandrake to the apple. Many other names, such as [“dog’s apple”] and [“devil’s apple”] are assigned to other categories.

#### Similarity to other plants

Although the mandrake is a well-known and very distinctive plant, sometimes its name refers to another plant. This could be because the other plant is better known locally, usually because it offers big, edible fruits (such as names related to apples, tomatoes, or even loquat). Such names are, e.g., [“wild tomato”] in Italian, [“eggplant”] in Spanish and Arabic, and [“earth loquat”] in Turkish. Conversely, we should mention here that the name “mandrake” has been also used for other plant species (see “Introduction” section).

#### Gender distinction

Our results also show that after taking into consideration the opinions explained in the introduction, the distinction between the male and female mandrake, made in ancient times, seems to have been a mental, intellectual, or scholarly construction. No true vernacular names allude to the gender of mandrake plants.

### Names related to the plant’s uses

#### Medicinal properties

Mandrake (*Mandragora* spp.) is perhaps the most famous medicinal plant in western culture since biblical times and throughout written history. This view has been clearly expressed by several authors, with statements such as: “Of all the medicinal herbs used in the ancient and medieval world, none was regarded with as much fear or wonder as the mandrake” [234: 189]). Harrison [171; 91] mentions that the plant’s medicinal virtues appear to have been discovered at a remote point in the development of ancient cultures, and although magical and superstitious beliefs tended to accompany its use, it seems clear that the narcotic qualities of the mandrake were appreciated through the entire period of its usage. Therefore, it is not surprising that “there is no medicinal plant known around which cluster more mysterious and quaint associations than around mandragora” [235: 6]. Mandrake was considered a panacea, especially in the Middle Ages [236]. In Wright’s [237: 182] words, “Mandrake is a cure for all except death.”

Thus, it is not at all surprising to find eleven names in seven languages that reflect the importance of the mandrake as a panacea down the generations. It is noteworthy to mention the names [“father of health”] in Arabic and Turkish, and [“doctor’s plant”] in German. The Armenian name [“king of all grasses”] deserves special attention. Arakelova [101: 153] mentions that in the Armenian folk tradition, the mandrake is “the king of all plants.” Its obvious sacred meaning among Armenians and its high estimation as a real panacea is proved by the existence of a special prayer-praise called Aγot ‘k ‘ *vasn loštakin* [“the Prayer to the mandrake”] [238: 285–286]; “You are the king of all the plants!/Almighty God created you and endowed you/With talent the of healing of people and nations/From all known illnesses in the name of the invisible and eternal God….”

Three names of the mandrake indicate specific medicinal uses of this plant. The Greek names *fistulóriza* and *fistulóchorto* mean [“fistula-healing root”] a use already mentioned by Hippocrates, *Fistulae*.11 [239: 822]; 4th–3rd BCE. The Turkish name *kankurutan* [“blood dryer”] reflects this use as indicated from Syria–Iraq in the 12th c. [144, II: 334] to “flow of the blood” and from Turkey [240: 2011] to “stops fever of the bile and blood.”

#### Narcotic, hallucinogenic, poisonous

Hyoscyamine and scopolamine (or hyoscine) stand out among the major tropane alkaloids in mandrake [241], although several other alkaloids are present [231, 242–246]. These tropane alkaloids are antagonists of muscarinic receptors (thus, with parasympatholytic effect), and have been described as having psychedelic and hallucinogenic properties. In higher doses, they may cause chronic spasms, a strong heartbeat, tachycardia, dilation of the pupil, inhibition of salivation, respiratory arrest, and coma. Therefore, mandrake was regarded as extremely hazardous to the level of being a mind-altering substance [241].

These widely known effects (narcotic, hallucinogenic, and poisonous) are reflected in 21 names in eight languages. Out of these, six names are related to the somniferous effect of the plant and eight to madness. The rest are related to poisoning, intoxication, and the causing of death.

#### Aphrodisiac

The reputation of mandrake as an aphrodisiac first appeared in biblical times (Genesis 30: 4–16). It was noted by Theophrastus in the 4th–3rd c. BCE [61, IX, 8. 8] and was widely expanded and iconographically depicted and transmitted in the medieval period, with reference mainly to the fruits [24]. Benítez et al. [13] found that this use was popularized in the last century and remained in the tradition with limited citations throughout history. A possible explanation has already been pointed out: “the fruit of the mandragora became a symbol of love” [247: 90], but not for actual use. Hanuš et al. [243] found no fewer than 136 chemical compounds in the mandrake fruit, but none of them is known to have an effect on human sexual behavior. They conclude: “The main compounds found in the ripe fruit and undetected in the unripe fruit are likely to be responsible for the fruit’s special taste and odor and its so-called aphrodisiac qualities.” Therefore, although mandrake’s aphrodisiac properties have never been pharmacologically proven, still today the fruit is eagerly sought for this purpose throughout its distribution range (especially in the south-east Mediterranean and the Balkans; [13]). Nevertheless, the profound of the Bible as well as local traditions are so deep rooted in many cultures that no less than 17 names in ten languages are related to love (especially “love apple or berry” (3) and “love plant or herb” (5), or other love-related attributes (e.g., [“the fruit that gets the lovers close”] and [“the dependent bride”] in Arabic). The relatively low number of names relating the mandrake to love and as an aphrodisiac (category C3; only 5.7% of total) reflects its rather rare use for this purpose down the generations. We note that many of the names related to the use of the plant come from ethnobotanical sources with field data from interviews with local people, or compendiums of these sources. Mentions of the plant, its names, and the different popular uses of it are not scarce in ethnobotanical works focusing on areas where it is native (e.g., [72, 129, 131, 167, 173] and references therein).

### Names related to the plant’s mythology

#### Black magic—sorcery, witchcraft, and magic, bad luck, and evil eye

Mandrake is probably the most celebrated of all “magical” plants in history and has thus given rise to a mammoth literature [12: 161, 248: 110, 249: 94]. The magical powers ascribed to the mandrake, both benign and evil qualities, made it an object of awesome veneration. In the folk imagination, the mandrake was conceived as a being with obvious ties to underworld forces [100: 105, 101: 153]. In many cultures, the mandrake has a notorious reputation as a plant used in black magic and witchcraft [250: 532]. In the Middle Ages, the mandrake was an indispensable element in the witch’s cauldron [44: 112, 109: 71]. Because the root has an uncanny resemblance to human limbs, the mandrake was considered half demon [251: 3], with great magical properties [252: 71].

The mandrake contains narcotic and hallucinogenic compounds which have the ability to heal, to injure, to cause madness, to induce a shamanistic trance, or to kill [117, 171, 179, 253], as is the case with other narcotic magical plants [27, 254, 255]. The ultimate result is that mandrake has a role in magic [142, 200, 235] and witchcraft [179, 256:166], especially during the Middle Ages [77]. Most famous was its use in witches’ flying ointment during the Middle Ages [29, II:342, 182, 257:166, 257:passim]. Witches’ activities are inseparable from demonic/devilish/Satanic worship; thus, the distinction between names related to witchcraft and those related to the devil/Satan is made from a purely technical viewpoint.

In our analysis, we recognize three subcategories related to this wide issue. Thirteen names in eight languages were found related to black magic and witches and witchcraft such as [“sorcerer’s root”] in English and Russian and [“magic root”] in Estonian, Dutch, and German. Four names are related to witches: [“witches’ herb”] in Dutch, English, and German, and [“witches’ love root”] in Dutch. All these names related to witches are from European countries which are beyond the natural distribution of the mandrake.

#### Evil supernatural elements—satan, devil, genie, monster, and dragon

Hildegard von Bingen (1098–1179 CE) stated: “The mandrake takes on and holds the influence of the devil more than other herbs because of its similarity to a human” [258: 33]. According to Thompson [251: 66–67], the narcotic effect of the mandrake, which may also cause death, is the reason for the plant’s association with the devil or an indwelling demon. In some places (such as England), it was still believed in the nineteenth century that the devil/Satan was perpetually looking on [180: 60].

Many authors discuss the relation between the mandrake and the devil/Satan [29, II:346, 33, 259]. The mandrake legend was at its height during the fifteenth, sixteenth, and seventeenth centuries, a period during which belief in the devil acquired an intensity and an immensity of scope unknown before or since [122: 268].

Twenty-eight names in nine languages were connected to various supernatural agents due to the narcotic and hallucinogenic effects. Nine names include the devil (e.g., [“devil’s plant”] in Dutch, [“devil’s apple”] in English, German, and Hungarian; [“devil’s food”] in English and Dutch); eight are related to various demonic Muslim figures (mainly *goula, jinn*), to Satan (e.g., [“Satan’s testicle”] in English, [“Satan’s apple”] in German, [“Satan’s turnip”] in Turkish), and three names include the word dragon: [“dragon’s apple”] in Dutch, [“dragon’s puppet”] in German, and [“dragon’s doll”] in English. Six names refer to various other evil agents, (e.g., [“little imp-man”], [“demon’s root”], and [“demon’s herb”] in German, [“goblin”] in Greek, and [“ghost’s apple”] in Turkish). It is noteworthy that while black magic and witchcraft names are restricted to Europe, the various other demonic figures appear in almost all the distribution range of the mandrake, probably as a result of its narcotic effect that was considered to be caused by supernatural evil, in addition to the “demonic” human-like root shape.

#### White magic—good luck, talisman, and dolls

The mandrake root was carved into human-like puppets or dolls which were highly regarded as omnipotent talismans [29, II:343–344, 56: 54–64, 122:26, 200:406, 201: 126–127]. The virtues ascribed to these dolls are not always the same: some act as love charms, others make the wearer invulnerable. But they all have two properties in common. They reveal treasure hidden underground and relieve their owner of chronic disease [122: 267]. These dolls were carefully treated, dressed in expensive clothes, fed (with food and wine), and were kept in special boxes with great tenderness [29, II:343–345, 215, 4:1674, 260, II:726, 261, III:487]. Germans formed little idols from mandrake roots and consulted them as oracles [180: 293]. In German folklore, mandrake came to be identified with the *alrune*, a devilish spirit and a magic root in human form who, when questioned, reveals all secret things touching welfare and increases possessions, enriches, removes all enemies, brings blessing on wedlock, and doubles every piece of coin laid under her [122: 262].

Few names are related to the positive fabulous powers of the mandrake doll/root which were used in white magic. According to De Cleene and Lejeune [29, II: 343], the German names *geldmännchen* ([“money manikin”]) and *glücksmännchen* ([“good luck manikin”]) reflect its ability to double money [29, 215, I:94, 262: 282]. Two other German names, *hausväterchen* [“little house father”] and *hinzelmannchen* [“gnome”] refer to a legendary creature resembling a tiny old man who lives in the depths of the earth and guards buried treasure; a gnome, while [“house’s father”] in German denotes the mandrake as a keeper of good luck. The French name *plante qui chante* [“singing plant”] even while not explicitly mentioning a doll is also related to the miraculous positive powers of the mandrake. In France (19th c.), the mandrake was thought to have the ability to sing, and its song had the magical potential to endow the alchemist with powers of transformation…just like the philosopher’s stone [263: 240–241].

#### Pulling-out ceremonies and supernatural phenomena

The andromorphic shape of the mandrake’s root caused it to be considered a semi-demon; thus, it screams while being pulled out and may cause death. This is the reason why a dog was used and why there was the need to close the ears [251: 3]. The pulling out of the mandrake, which is related to its supernatural evil powers, is threatened by revenge from these powerful agents [180, 201: 124–125, 210: 17–18, 251: 153]. This is the very reason why special ceremonies needed to be performed before and during the digging out of the mandrake to pacify these elements. Otherwise, they might immediately kill the herb collectors (“rhizotomies”) if they did not take the proper precautions to avoid the expected dangers as stated above. The main elements of the ceremony are special incantations, prayers, and dances, and the use of a dog.

The first to mention the use of a dog while pulling mandrake is Flavius Josephus (first c. CE), provided that the plant name he mentions, “*ba’aras,”* really is the mandrake, as it is agreed to be by most authorities (e.g., [186, III: 368]). According to Josephus, *Jewish War* VIII:6.3 [264], the dog dies immediately. The dog is mentioned again in relation to mandrake pulling-out ceremonies in Iran in the sixth c. [111: 2], Syria, twelfth c. [144, II: 708–710], Uzbekistan (today’s borders), thirteenth c. [41: 116], Andalusia, thirteenth c. (under the name [“dog killer”] [125]), Hindustan, fourteenth-fifteenth c. [154, 6, footnote 4], Dagestan, sixteenth c. (possibly associated to *M. turcomanica*) [198: 409], Armenia, eighteen c. [265: 99] and again in Iran [266: 13] (period not given). The dog was used as a scapegoat to avoid the danger of immediate death caused by the voices that the plant produces. The Avars of Dagestan (northern Caucasus, Dagestan) call this herb *xIapuleb xer* [lit. “barking grass,” “grass (causing) barking”] [184: 1486], and call their witches simply *xIapulel ruččabi* [“barking women”], since the witches bark during the digging ceremony [102: 251–2]. In the Yezidi folk tradition, to avoid death, the soil must first be dug around the root, after which a hungry dog or a goat is tied to it [101]. The first pictorial reference linking the plant to the dog appears to be in the 6th c. in Europe: the illustration of Dioscorides with the nymph Heuresist in the so-called Vienna Dioscorides or Juliana Anicia Codex [142: 69] and later only from the 12th c. onward [142: 69, 201: 380]. Our survey includes 15 names in nine languages which relate mandrake with a dog. Two names ([“dog’s apple”] in Dutch, French, German, Greek, Italian, and Turkish and [“dog’s testicle”] in Turkish) include the word "dog," while four ([“dog killer”] in Arabic, [“dog uprooter” = dog killer], [“dog killer”], and [“dog breaker”] in Farsi) explicitly express the fate of the dog in the ceremony. While the general names are mainly from Europe, the four specific names are in Arabic and in Farsi. This list, combined with the literature on the role of the dog in the ceremony from Iran and adjacent countries, strongly sustains the view that the use of a dog in this ceremony originated in this region.

It is commonly stated in the literature that when being pulled out, the mandrake will let out a scream and the dog will die [54: 46, 251: 168–170]. An erudite analysis by Van Arsdall et al. [142: 317] shows that the screaming element only reached Europe from the twelfth c. In the first record of a mandrake pulling-out ceremony in the Middle East from Flavius Josephus [264: VIII:6. 3] (1st c. CE, Judea), there is no mention of a scream. A Syrian-Jewish story composed in Damascus in the twelfth–thirteenth centuries [215: 14] mentions the scream. The belief in the screaming mandrake still exists in and around Iran. For example, in northwest Iran there is a belief even today that the mandrake “groans” [267: 54–65]. In Armenia, in the eighteenth century, it is mentioned that the mandrake “moans with a human voice” [265: 99]. In the Yezidi folk tradition, the mandrake supposedly shouts so shrilly that one who digs it up dies at once [101:153].

We found seven names related to the scream (Tables 5 E1, 9), all of which are from Europe, particularly Poland (3 names), where mandrake was widely used in witchcraft to prepare flying ointment, mainly during the sixteenth-seventeenth c. [182: 180]. Mandrake does not grow in Poland, and both the term and the folklore attached to *M. officinarum* have been applied to local herbs, usually *Atropa belladonna*, deadly nightshade [12: passim, 182: 194–195]. However, it is not at all clear why mandrake was so tightly related to the element of the scream in Poland. The first records about the screaming mandrake appear simultaneously in Europe and the Middle East in the twelfth c. The prevalence of this belief in northwest Iran and Armenia nowadays may serve as indirect evidence for its origin in this part of the world. Rahner [199: 214] assumes that the scream was imported from Arabic or eastern sources without giving any references.

**Table 9:** Mandrake name distribution according to languages and categories**:** 292 vernacular names for the plant in 41 languages

Category	A			B			C			D			E			F		G NC			Total
Subcategory/	A1	A2	A3	B1	B2	B3	C1	C2	C3	D1	D2	D3	E1	E2	E3	F1	F2	G1	G2	NC	
Language																					
Albanian	1																				1
Arabic			3	1	3	3	2		4		9			1	2			1		3	32
Aramaic			1	1																	2
Armenian	1				1		1											1			4
Basque																				1	1
Bengali			1																		1
Berber																				2	2
Bulgarian	1																				1
Catalan	1																			1	2
Chinese																				1	1
Corsican	1																				1
Czech				2							1										3
Dagestani														1							1
Danish		1		1					1												3
Dutch	2	2		5				3		3	3			1		4					23
English	1			3				3		3	4							2	1		17
Estonian										1											1
Farsi			1	1	1				1					3							7
Finnish		1							1												2
French	1			2		2				1		1	1	1		1		1			11
Gaelic								1													1
Georgian	1																				1
German		1		7		3	1	5	3	2	6	4		1		7		2			42
German,Old							1	1	1												3
Greek	2			3	5	5	2	6	1		1			1				1		8	35
Hebrew									1												1
Hungarian	1							1		1	1										4
Icelandic																1					1
Italian	1													3		1					5
Latin				2		2														1	5
Norwegian		1																			1
Polish	1			1							1		3							1	7
Portuguese	1																				1
Russian	1									1			1		1		1				5
Serbo-Croatian	3	1			4	1	1			1			1				1			1	14
Slovak						1															1
Spanish	2				1	7		1												2	13
Syriac																				1	1
Swedish		2																			2
Turkish	1		2	4	3	4	3		3		2		1	3			1			4	31
Ukrainian	1								1												2
Total	24	9	8	33	18	28	11	21	17	13	28	5	7	15	3	14	3	8	1	26	292

Abbreviations for categories and subcategories as in Tables 1, 2, 3, 4, 5, 6, 7 and 8

*NC* non-classified names

Mandrake may shine and produce lights: the first to mention the burning mandrake was again Josephus Flavius [265, VIII:6.3] (we accept the general agreement that his plant named *“ba’aras”* is the mandrake; see [186, III:365]). In the Herbarium Apuleii Platonici, the author (4th c. CE; see [268: 39]) states: “it [the mandrake] shineth at night altogether like a lamp.” The Arabic name *sirag al kutrub* is usually translated as [“the devil’s candle”] [189, I:49, 190: 250]. Al Baithar [88: 14] wrote, “this medicine (the mandrake) is called *sirag al kutrub* and is like a torch at night … the cortex of the root (is like) fireflies which glow at night…it looks like a fire.” Richardson [189, I:49] explains: “The devil’s candle, on account of its shining appearance in the night, from the number of glow-worms which cover the leaves.” Another Arabic name, [“the burning mandrake”] corroborates this tradition. Roger [269: 47] visited the Holy Land in the 17th c. and reported that the mandrake emits sulphurous vapors and glows at night. Stories of the shining mandrake are frequently repeated by European sources (e.g., [142, 190, 250, 270:144, 271: 330]). Arakelova [101: 153] mentions that the Yezidis believe that at night mandrakes glitter and their leaves look like silver. So, this ancient belief still survives to this day in Iran and Armenia.

#### Thief, gallows, semen, and the mandrake

The belief that the mandrake grew under the gallows from the semen of hanged victims [142, 195:59–60] was first recorded in 1532 by the physician and botanist Otto Brunfels (1488–1534) and later became widespread in European medical literature during the seventeenth century [142, 201:121–122]. However, the gallows mandrake tradition was strongest in German lands [195: 60]. Talley [74: 166–168] relates the legend that mandrakes come from the urine or semen of a thief hanged on the gallows to sacrificial rites and myths of pre-Christian Germanic people. He finds parallels between Odin’s human sacrifice (in Nordic mythology) and the growth of the mandrake from urine or semen under the gallows, while Randolph [200: 495] connects it to the myth of Prometheus (see below). Starck [56: 79] already concluded that the belief in the mandrake as a magical plant with human form which grew from the semen of a sleeping or dead man had its origins in Mesopotamia or Persia (see [41: 115] for the same view), while according to van Arsdall et al. [142: 330]: “Its origin is not known at the present time.”

Another aspect of the mandrake-gallows connection is “the hand of glory.” The dried hand of a hanged man was believed to have magical properties and was used by thieves and burglars [272: 99–100]. The notorious hand of glory is known in French as the *main de gloire*, which is thought to be a corruption of the French for mandrake: *mandragore*. There is an obvious shared association with the gallows corpse [195: 62]. The myth arose among thieves and illiterate persons during the Middle Ages in France through a misunderstanding of words, *mandragore*, the French term for the mandragora or mandrake, being mistaken for *main de gloire*. The term *mandegloire* is given as a popular synonym for *mandragore* [273: 59].

All the names (14) related to gallows are from Europe, especially from Germany (7) and the Netherlands (4): it is logical to think that Europe must be the cradle of this myth. Examples are: [“gallows’ youngling”] and [“gallows’ man”] in Dutch; [“torturer’s root”], [“little gallows’ man”], [“executioner’s root”] and [“piss thief”] in German.

### Names related to religion and historical characteristics

#### Adam’s creation

Hildegard Von Bingen (twelfth c.) posits that the mandrake came forth from the same seed from which Adam was created, and to some extent its shape resembles that of man [258: 1151A-1152A]. According to a Persian myth, man was created from a plant that resembled a human shape; this plant is a rhubarb (*Rheum* spp., originally named “ribas”). The tradition says that Gayōmart, the first man, was created by Ahuramazda. When he died, there came a seed from his loins … (it) was kept inside the soil. After forty years, it changed into ribas (or a mandrake) [266: 12]). Eliade [274] has already pointed out the striking similarity between the myth of *Gayōmart* and the traditions about mandrake; the union of anthropomorphic plant and shining power which fertilizes the earth. *Gayōmart'*s seed creates a hybrid creature, partway between a human being, an animal, and a plant [275: 186]. Zarcone [41: 120] concludes: “mandrake follows in the tradition of all the myths that mention the birth of humans from the earth at the beginning of time.”

Despite these ancient Iranian myths concerning the creation of the first man and mandrake, which may have migrated very late to the West, there is no name in this language related to this issue. The Russian [“Adam’s head”] and the Turkish [“Adam’s plant”] may hint at a relic of the ancient Iranian myth. One might consider that the German legend has a negative connotation (spawn of evil), and all the eastern myths (as well as Hildegard von Bingen’s text [258]) have a positive one (creation of peoples).

#### King Solomon

The name [“King Solomon tree”] in Arabic and in Armenian is a relic of an old legend that there was a piece of a mandrake in the ring of King Solomon, who used it against demons. This story follows an old tradition handed down under the name of Hermes Trismegistos via Ibn al-Bayṭār [276, III: 14] (13th c.). According to Amasiaci [198: 96], in fifteenth-century Armenia, mandrake was called [“the tree of Solomon”]; it was thought to bring its owner happiness and power over people.

#### Circe

Dioscorides [45: 4.75] cites a specific plant called by the ancients “Circe’s root” (*kirkaía ríza/κιρκαία ρίζα*) and, according to Pliny in *Natural History* [277: 25.147], as *Ciecaeon*, referring to the passage in the *Odyssey* (10.210–43) in which Homer describes Circe, the mythical sorceress who turned men into sexually supercharged swine. Several authors have suggested that this enchanted plant was the mandrake [199: 228–229, 200: 501–505, 201: 114, 278: 204]. So, it is not surprising that this name persisted in English as “herb of Circe,” *Circée* in French, and *zauberpflanze der circe* [“Circe’s magic plant”] in German.

#### Prometheus

Apollonius of Rhodes (third century BCE [279:3,843-868ff]) reports: “Prometheus was condemned to his punishment for theft…the flower sprang from his gore as it dripped to the ground.” According to Randolph [200: 495–496], “since gore does not drip from the bodies of hang-thieves, a change had to be made here…in adapting the story to the mandrake, and so the plant is said to spring from the thief’s urine” (see also [29, II:338, 201: 121, 280:passim]). Van Arsdall et al. [142: 293] reject this view. It still might be the origin of the thief/gallows/semen/urine myth. The question is, what happened with the story from the third c. BCE to the fifteenth c. AD? The semen and urine of a hanged thief in connection with the mandrake legend is relatively late, entering written sources only about 1500 [195: 59–60]; one name in German ([“Prometheus plant”]) and the English name “herb of Prometheus” retain this old myth.

#### Paradise, mandrake, and the elephant

The mandrake’s rare name “elephant ear” [202: 249] is related to a medieval legend of the elephant and the mandrake. A Latin bestiary of the eighth or ninth century tells the tale of an elephant great in intellect but feeble in the desire to reproduce. The cure was for him and his mate to travel eastward until, near paradise, they found the mandrake plant [281: 248]. A male and female elephant (Adam and Eve) require the fruit of the mandrake (here, the “Tree of Knowledge”) to arouse their sexual desire [282: 153]. The German name [“little child’s root”] indicates that this is the plant that the elephants ate in paradise [31: 41].

### Non-classified names

Non-classified names constitute the fifth widest category (26 names; Fig. 2), indicating that a lot of names are of unknown origin despite all the above-mentioned connections between names and the plant’s properties, shape, or features. Languages such as Basque, Berber, Chinese, and Slovak all have names for the plant in this category, but ten more also have names in this group (Arabic, Catalan, Greek, Italian, Latin, Polish, Serbo-Croatian, Spanish, and Turkish). Some Greek names included under this section could represent euphemistic names. Euphemism has been in use since Greek antiquity as a kind of innocuous expression to describe or name something that is considered dangerous or unpleasant. By using bland, inoffensive terms, the speaker appears to treat the subject in a positive rather than pejorative way and avoids invoking its malign characteristics. In a way, euphemisms are used to politely refer to negative life issues such as disability, disaster, death.

### Analysis of hypotheses

Our hypothesis 1 suggests that due to the longer history and larger distribution area around the Mediterranean (especially in the Middle and Near East), it is expected to find migration of names (and myths and customs reflected in these names) from east to the west (Middle East to Europe) and from the south to the north (southern Europe to western and central and northern Europe) than migrations in the opposite directions.

Several authors [32: 261, 41: 115, 185: 200, 191: 89, 200: 499, 226: 442, note 1] consider Persia to be the original home of mandrake superstitions based mainly on the role of the dog (see below), with a linguistic background (the origin of the word “mandragora”; see above) that was disputed by Asatrian [100: 106].

Our data show that three Farsi mandrake names are related to the role of a dog in the pulling-out ceremony: *sag-kuš* (lit. [“dog slayer”], *sag-šikan* [lit. “dog breaker”], *sag-kan* [lit. “dog-dug”]). Two more names related to this issue occur in Arabic and Dagestani (also named Avar). The other mandrake names related to a dog (in five languages) could be translated as “the dog apple.” We assume that the mention of a dog in relation to the mandrake in western languages is a kind of echo of its role in the pulling-out ceremony in the Middle East (and Iran), since the use of a dog has deep roots in this region (see above).

As expected, we were unable to pinpoint the migration of any name from northern Europe to the south or from Europe to the Middle East.

It was expected (hypothesis 2) that we would find more names (and in more categories) in countries in which the mandrake is native (especially around the Mediterranean) and has a longer history than in other parts of Europe in which mandrake (and its legends) arrived later. The order of the languages according to number of mandrake names (Table 8) (not counting spelling variants) is as follows: German 45 (including 3 Old German ones); Greek 35; Arabic 32; Turkish 31; Dutch 23; English 17; Serbo-Croatian 15; Spanish 13; French 11, Latin 8, Polish 7, and Farsi 7. Since mandrake (*M. officinarum/autumnalis*) is a Mediterranean/Middle-Eastern plant, our hypothesis must be rejected. According to the distribution map presented by Volis et al. [23], only Arabic, Aramaic, Berber, Corsican, Greek, Hebrew, Italian, Latin, Portuguese, Spanish, Syriac, and Turkish are currently spoken in the areas were the plant naturally grows. Therefore, while 129 names in these 12 languages are used to mention the plants in the areas where they naturally occurs nowadays, 163 names are or have been used in other territories, and our hypothesis must be rejected. Concerning the proliferation of the mandrake’s German names, it seems that this reflects the importance of the mandrake in the local witchcraft and folklore in this country [251: 12, 283: 144]. Nevertheless, another cause of the highest records in languages as German can be a higher availability of written sources in these languages.

Since the mandrake has a long history as an aphrodisiac and as an omnipotent medicinal plant down the generations [13], it was expected (hypothesis 3) to find relatively many names related to these categories. In fact, we found (Table 3) relatively few names which express hallucinogenic or narcotic aspects (21 names, C2) or are related to “love issues” (17 names, C3), and even fewer related to its general medicinal properties (11 names, C1). Thus, just 17% of total names are related to this category (Fig. 2), and our third hypothesis must be rejected.

It was expected (hypothesis 4) that we would find more names related to witchcraft and black magic in Europe than in the Muslim world. Thompson [251: 131] has already mentioned that “In Germany in medieval times belief in the powers of the mandrake became a universal cult, and through the country the plant was regarded with veneration for its magical properties.” Names related to this category comprise nearly 16% of the totals (Table 8, Fig. 2). Of the 46 names (Table 4 D1–D3), except for nine in Arabic, and two in Turkish, the rest occur in European languages. Thus, this hypothesis was confirmed.

## Conclusions

Mandrake has been named in diverse ways since antiquity, summing up to 292 vernacular names in 41 languages. Vernacular names reflect local manners, beliefs and uses, many of which wandered during history. The distribution of mandrake’s vernacular names according to the designated categories reflects its widespread historical reputation as related to the doctrine of signatures, beliefs in its supernatural powers, mystic beliefs, and to a lesser extent its uses in magic and medicine. Most of the vernacular names are related to the plant's morphology (79 names, B1–B3), rather than its pharmacological (49, C1–C3) or mystical (46, D1–D3) properties, the pulling out ceremony (25, E1–E3) or other aspects.

Van Arsdall et al. [142] have already noted that different myths related to the mandrake have different origins and ages. Some are ancient while others are later, according to the written evidence. This observation could be examplified in several cases as inferred from spatiotemporal analysis of the mandrake names. The pulling-out ceremonies for the mandrake (especially with the use of a dog) originated in the Middle East. In this category we have 25 names, 28% of them from the Near East. The scream heard during the pulling out is a later phenomenon [142: 317] and is reflected in four European languages and in Turkish.

The relation of the mandrake’s names to magic and witchcraft appears only in European countries in which the mandrake is non-native (Table 4 D1). This is also indirect evidence for a later expansion of these beliefs after the introduction (or import of the roots) of the mandrake to these countries. Names which are related to demonic agents (as a result of the similarity to the human shape) appear mainly in Arabic but also in seven European languages, most of them from countries in which the mandrake is not indigenous. This finding may provide indirect support for Middle-Eastern origin of this aspect. Thus, it seems that witchcraft and magical traditions originated mainly in central Europe (see above), while the fear of the plant due to its shape may have an eastern origin.

The origin of the mandrake under the gallows has late, western roots. All the names related to gallows are from Europe (14), especially from Germany (7) and the Netherlands (4) and for sure reflect a late-Medieval European origin.

## Data Availability

Not applicable.
