# Correction: In search of traces of the mandrake myth: the historical, and ethnobotanical roots of its vernacular names

**DOI:** 10.1186/s13002-024-00688-7

**Published:** 2024-05-13

**Authors:** Amots Dafni, Cesar Blanché, Salekh Aqil Khatib, Theodora Petanidou, Bedrettin Aytaç, Ettore Pacini, Ekaterina Kozuharova, Aharon Geva-Kleinberger, Soli Shahvar, Zora Dajic, Helmut W. Klug, Guillermo Benítez

**Affiliations:** 1 Department of Evolutionary and Environmental Biology and Institute of Evolution, University of Haifa, Haifa, Israel; 2 GREB-BioC, Botany Laboratory, Faculty of Pharmacy and Food Sciences, University of Barcelona, Av. Joan XXIII S/N, 08028 Barcelona, Catalonia Spain; 3 20128 Mghar, Israel; 4 Laboratory of Biogeography and Ecology, Department of Geography, University of the Aegean, 81100 Mytilene, Greece; 5 Department of Arabic Language and Literature, Faculty of Languages, History and Geography, Ankara University, Ankara, Turkey; 6 Department of Life Sciences, Università Degli Studi Di Siena, Siena, Italy; 7 Faculty of Pharmacy, Department of Pharmacognosy, Medical University of Sofia, Dunav 2 Sr., 1000 Sofia, Bulgaria; 8 Department of Arabic Language and Literature, University of Haifa, Haifa, Israel; 9 Department of Middle Eastern and Islamic Studies, The Ezri Center for Iran and Persian Gulf Studies, The University of Haifa, Haifa, Israel; 10 Faculty of Agriculture, Department of Applied Botany, University of Belgrade, Nemanjina 6, 11080 Belgrade, Republic of Serbia; 11 Centre for Information Modelling, University of Graz, Graz, Austria; 12 Department of Botany, University of Granada, Campus Universitario de Cartuja, 18071 Granada, Spain

**Correction : Journal of Ethnobiology and Ethnomedicine 2021, 17(1):68** 10.1186/s13002-021-00494-5

Following publication of the original article, an error in the author list was brought to the attention of the journal: the family name of the seventh author, Ekaterina Kozuharova, had been misspelled as ‘Kohazurova’. The article has since been corrected. The authors thank you for reading this erratum and apologize for any inconvenience caused.

